# Supplementary material for: Developing Nanodisc-ID for label-free characterizations of membrane proteins
Source: Commun Biol. 2021 Apr 30;4:514. doi: 10.1038/s42003-021-02043-y (PMC8087782; doi:10.1038/s42003-021-02043-y)
Supplement: Supplementary file 1 — Supplementary Information [file 42003_2021_2043_MOESM1_ESM.pdf]

Figure S1

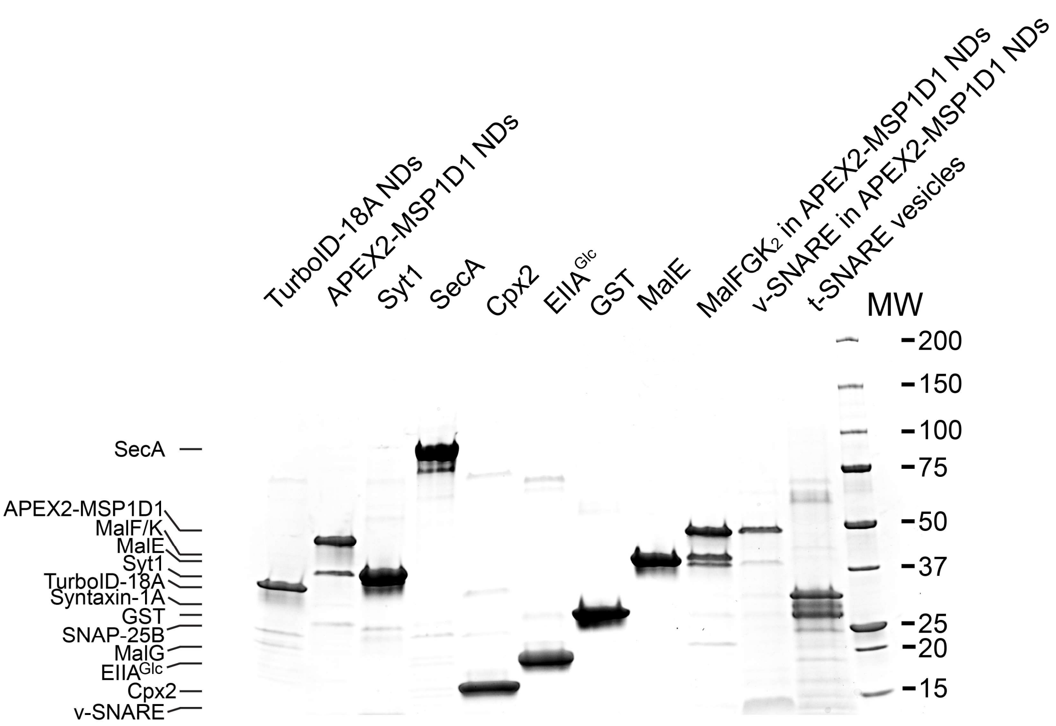

Figure S1. SDS-PAGE of protein samples used in this study.

Proteins were subjected to denaturing gel electrophoresis and stained with Coomassie Blue. TurboID-18A NDs and APEX2-MSP1D1 NDs were both made of PI lipids. MalFGK$_2$ NDs, v-SNARE NDs and t-SNARE vesicles were made of PC lipids.

Figure S2

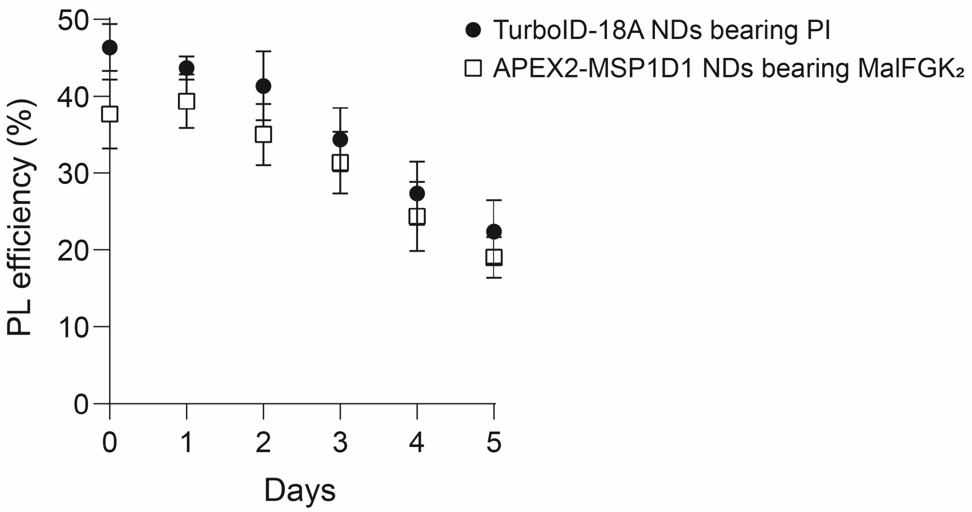

Figure S2. Stability of the nanodisc-ID approach.

TurboID-18A NDs bearing PI and APEX2-MSP1D1 NDs bearing $MalFGK_2$ were kept on ice for the indicated days, and then used to bind and label syt1 and MalE, respectively. The percentages of labeled syt1 and MalE were quantified and plotted. Data are shown as mean ± s.d., n = 3 independent experiments.

Figure S3

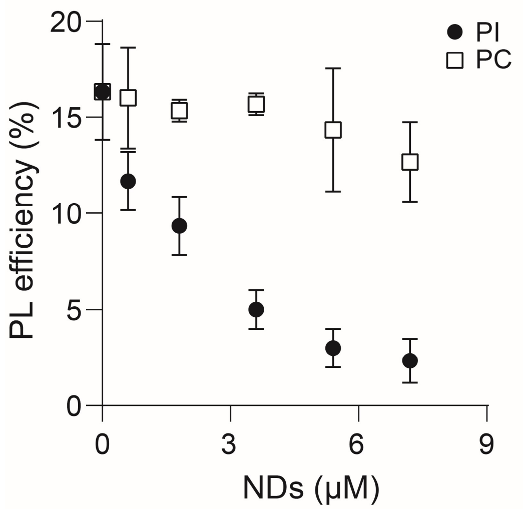

Figure S3. Analysis of competitive binding of PS and PI to syt1 using nanodisc-ID.
Syt1 were incubated with TurboID-18A NDs bearing PS and MSP1D1 NDs bearing PC or PI at the indicated concentrations at room temp, followed by PL reactions. The percentages of labeled syt1 were quantified and plotted. Data are shown as mean ± s.d., n = 3 independent experiments.

Figure S4

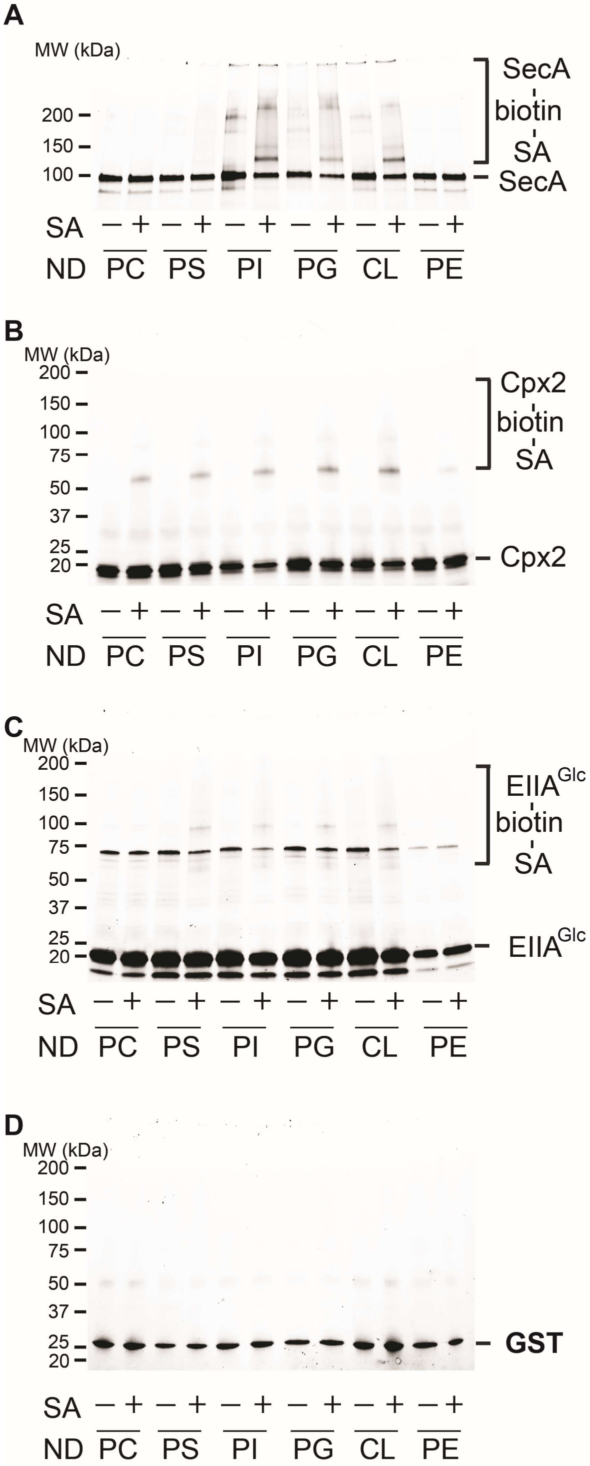

Figure S4. Representative gel images of pMP-lipid interactions characterized using the nanodisc-ID approach. The decreased intensities of each protein upon incubation of SA were quantified in Fig. 2A. The pMPs shown here are SecA (**A**), cpx (**B**), EIIA^{Glc} (**C**). GST (**D**) was used as a negative control.

Figure S5

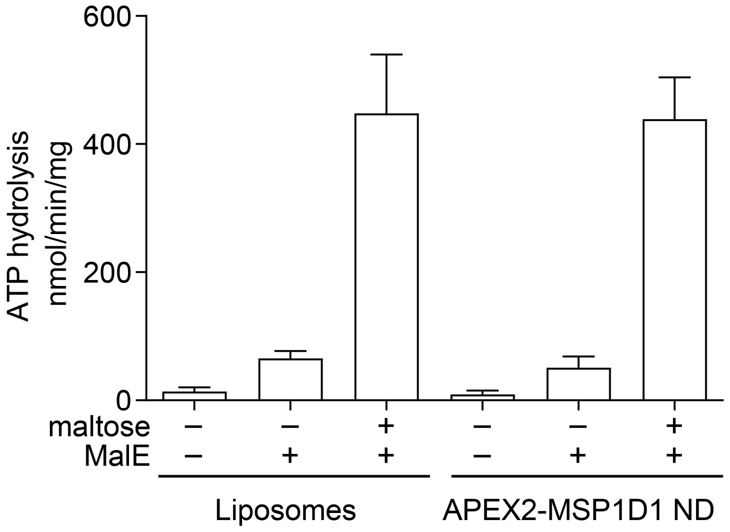

Figure S5. Functional reconstitution of $MalFGK_2$ in APEX2-MSP1D1 NDs.

ATPase activities of $MalFGK_2$ reconstituted in APEX2-MSP1D1 NDs or liposomes. ATP hydrolysis was measured in the presence or absence of MalE and maltose. Data are shown as mean ± s.d., n = 3 independent experiments.
