# Supplementary material for: Developing Nanodisc-ID for label-free characterizations of membrane proteins
Source: Commun Biol. 2021 Apr 30;4:514. doi: 10.1038/s42003-021-02043-y (PMC8087782; doi:10.1038/s42003-021-02043-y)
Supplement: Supplementary file 3 — Description of Additional Supplementary Files [file 42003_2021_2043_MOESM3_ESM.pdf]

Description of Additional Supplementary Files

File name: Supplementary Data 1

Description: Source data for Figs. 3 and 5.
